# Y/X-Chromosome-Bearing Sperm Shows Elevated Ratio in the Left but Not the Right Testes in Healthy Mice

**DOI:** 10.3390/life11111219

**Published:** 2021-11-11

**Authors:** Chengqing Hu, Jiangcheng Shi, Yujing Chi, Jichun Yang, Qinghua Cui

**Affiliations:** 1 Center for Noncoding RNA Medicine, Department of Physiology and Pathophysiology, Department of Biomedical Informatics, MOE Key Laboratory of Cardiovascular Sciences, School of Basic Medical Sciences, Peking University, 38 Xueyuan Rd, Beijing 100191, China; Hcq@bjmu.edu.cn (C.H.); sjc@bjmu.edu.cn (J.S.); 2 Department of Central Laboratory & Institute of Clinical Molecular Biology, Peking University People’s Hospital, Beijing 100044, China; chiyujing@bjmu.edu.cn

**Keywords:** X-chromosome-bearing sperm, Y-chromosome-bearing sperm, sex chromosome

## Abstract

The sex chromosomes play central roles in determining the sex of almost all of the multicellular organisms. It is well known that meiosis in mammalian spermatogenesis produces ~50% Y- and ~50% X-chromosome-bearing sperm, a 1:1 ratio. Here we first reveal that the X-chromosome-encoded miRNAs show lower expression levels in the left testis than in the right testis in healthy mice using bioinformatics modeling of miRNA-sequencing data, suggesting that the Y:X ratio could be unbalanced between the left testis and the right testis. We further reveal that the Y:X ratio is significantly elevated in the left testis but balanced in the right testis using flow cytometry. This study represents the first time the biased Y:X ratio in the left testis but not in the right testis is revealed.

## 1. Introduction

The sex chromosomes play central roles in determining the sex of almost all multicellular organisms [1]. In mammals, there are two types of sex chromosomes: the X-chromosome and the Y-chromosome. The sex of a baby is determined by either a Y- (male) or an X-chromosome (female) inside the father’s sperm that successfully fertilizes the mother’s egg. The origin, maturation, and functions of the two sex chromosomes are reported to be mostly identical and no major differences exist between the two sperm types except their DNA [2]. Moreover, according to Mendelian segregation, it is also well known that meiosis in mammalian spermatogenesis produces ~50% Y- and ~50% X-chromosomes, a 1:1 ratio [2,3].

More recently, however, it was reported that the balance of the Y:X ratio was shifted under specific genetic and environmental conditions [2,4]. For example, stressful conditions increased the expression of apoptotic proteins, which then led to an increase in the Y:X ratio [5]. Lavoie et al. reported that male-dominated environments produced elevated levels of Y-chromosome-bearing sperm (Y-CBS) in wild house mice [6], whereas Firman et al. reported an opposite tendency, in which male–male competitive ‘risk’ produced lower proportions of Y-CBS [7]. It was revealed that male Peromyscus leucopus with high levels of genetic variation tend to have ejaculates with a higher Y-CBS proportion [3]. Small deletions in Sly-related regions can result in a higher ratio of X-CBS [8]. Exposure to organochlorine pollutants increased the ratio of ejaculated Y-CBS [9]. In adult Inuit men, a negative trend between the Y:X ratio and serum concentration of perfluorooctanesulfonate was observed [10]. Although a number of studies reported a Y:X ratio difference in animals and humans, the difference is normally quite small and was considered to have little or no biological significance [2].

We had previously identified a number of sex-biased genes and microRNAs (miRNAs) and revealed that they show significant bias in biological functions, disease risks, and therapeutics [11,12,13]. Moreover, biased miRNAs and functions between the left and the right testes in healthy male mice were also revealed using miRNA sequencing and qRT-PCR [14]. Given the context of the Y:X ratio balance, it is thus important and interesting to investigate whether the Y:X ratio changed between the left and the right testes, especially whether there is difference in the Y:X ratio between the left and the right testes. To address these issues, we first predicted a higher Y-CBS/lower Y-CBS in the left testis than in the right testis using a bioinformatics model and then confirmed this finding by flow cytometry. More interestingly, we further reveal that the sex-chromosome imbalance between the left and the right testes results in the left testis but not the right testis, as confirmed by flow cytometry in healthy mice on a background of C57BL/6J. That is, we uncovered that Y-CBS showed a higher proportion than X-CBS in the left testis, whereas the Y:X ratio is almost 1:1 in the right testis.

## 2. Materials and Methods

### 2.1. Prediction for Y:X Balance between the Left and the Right Testes

We first obtained the miRNA expression dataset of the left and the right testes from three healthy mice from our previous study, which were quantified based on the unique molecular identifier (UMI). We then located the miRNAs in the X-chromosome of mouse genome from miRBase Database (version 22.1) [15] and then defined these miRNAs as the X-miRNA set, a miRNA set consisting of all X-chromosome encoded miRNAs (169 miRNA members). Finally, the two-phenotype miRNA set enrichment analysis (tpMSEA) tool in the sTAM software [16] was used to evaluate whether the expression of these X-miRNAs is biased to the left testes, to the right testes, or balanced between the left and the right testes.

### 2.2. Experimental Mice

Eight- to ten-week-old healthy male mice on C57BL/6J background (weight 22–25 g) were used in this study. All animals were bred and housed locally at 24 ± 2 °C and were acclimated to standard laboratory conditions (12L:12D cycle) with free access to rodent feed and water. All procedures involving experimental animals were approved by the Institutional Animal Care and Use Committee of Peking University Health Science Center.

### 2.3. Sperm Cell Isolation and Staining

A percentage of 4–5% isoflurane was used to induce anesthesia, and 1%–3% isoflurane was applied to keep the mice anesthesia in the whole operated process. The left and right epididymis were removed respectively, and then the caudal epididymis was excised and transferred to a dish containing 2 mL 37 °C prewarmed Enriched Krebs-Ringer bicarbonate medium (EKRB, G0430, Solarbio Science & Technology, Beijing, China) [17,18,19] with 2% FBS, and immediately cut into 4 pieces to fully release the mature sperms into the medium. After 10 min, the sperm suspension was filtered through 40 μm diameter strainers to remove small clumps and then evenly and gently transferred and stained with Hoechst 33,342 (5 μg/mL, C0031, Solarbio Science & Technology, Beijing, China) for 45 min at 35 °C in the dark, gently mixing to keep the sperm equalization every 15 min.

### 2.4. Flow Cytometry

Many techniques have been used to separate mammalian X- and Y-bearing sperm through the past decades, mainly including the H-Y antigen separation method [20], density gradient centrifugation method [21,22], electrophoresis method [23], protein immunological separation method [24,25,26], flow cytometry [27], etc., all of which focus on physical or immunological separation. However, those procedures have not been scientifically proved to be effective, resulting in unconfirmed separation followed by inconsistent birth ratio results, except for flow cytometry. At present, flow cytometric sperm sorting based on X and Y sperm DNA differences has been confirmed as the most effective method, and its separation accuracy is more than 90% [28,29]. Based on the DNA content of X-CBS, which is 3.2% higher than that of Y-CBS in mice [30], the fluorescence signal of X-CBS is stronger than that of Y-CBS. When sperms pass through the flow cytometer, the fluorescence signal of X-CBS is stronger than that of Y-CBS, so the detectors could identify the types of sperm well [28,31,32]. Fully stained sperms were analyzed, and at least 20,000 sperms were randomly selected each time for detection. BD FACSymphony^TM^ S6 System (BD Biosciences, San Diego, CA, USA) and FlowJo software (version 10.4, BD Biosciences, San Diego, CA, USA) were used for data acquisition and analysis. The data are shown as the ratio of X- or Y-CBS from the left or right caudal epididymis.

### 2.5. Statistical Analysis

The results are presented as the mean ± SEM (N = 7). Experimental groups are compared using the two-tailed unpaired Student’s *t*-tests. *p*-values of <0.05 are considered statistically significant.

## 3. Results

### 3.1. X-miRNAs Showed Significantly Lower Expression in the Left Testis

**Figure 1:**
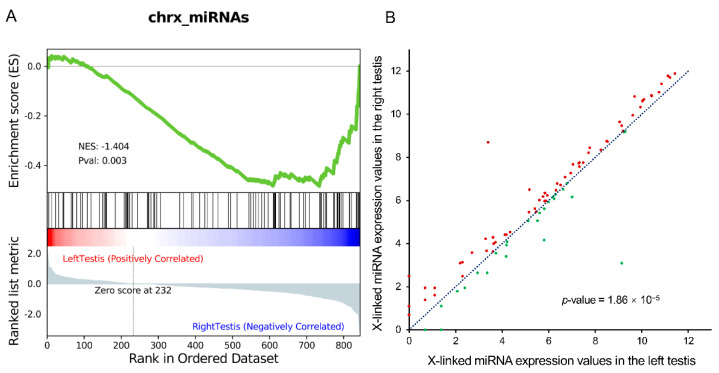
Comparison of X-chromosome-encoded miRNAs (chrx_miRNAs, X-miRNAs) expression in the left and right testis. (**A**) Enrichment analysis result of the miRNA expression data from the left and the right testes of healthy mice in the X-miRNAs. First, the miRNA expression difference between the left and the right testes was measured by a statistic (here we used signal-to-noise ratio), then the miRNAs were ranked from high to low according to the statistic. The black lines in the intermediate vertical bands mark the positions of the X-miRNAs mapped in the ranked list. The bottom graph shows the miRNA expression differences between the left and right testes. The green line in the upper graph indicates the enrichment degree of X-miRNAs between the left and right testes and finds that X-miRNAs are enriched in the right testes according to the enrichment score, suggesting that the Y: X could be unbalanced between them. (**B**) Expression-based comparison of X-miRNA expression under the logarithmic coordinates. The red points indicate the X-linked miRNAs with higher expression levels in the right testes, while the green ones indicate the opposite. The blue line represents the diagonal line (equal expression in the two sides of testes). Wilcoxon signed-rank test, *p*-value < 0.05.

Interestingly, the sTAM software revealed a more significant enrichment of the X-miRNAs in the right testis than in the left testis (enrichment score = −0.48, normalized enrichment score = −1.40, *p*-value = 0.003, Figure 1A). This finding means that the expression of the X-miRNAs is lower in the left testis than in the right testis, which further suggests that Y-CBS could have higher proportion in the left testis than in the right testis. Moreover, we also compared the X-miRNA expression values of the left and right testes directly, and the Wilcoxon signed-rank test confirmed that the miRNA expression values of the left testes were significantly less than those of the right (*p*-value = 1.86 × 10^−5^). Statistically, most of the miRNAs (65 vs. 27) exhibited higher expression levels in the right testis than those in the left testis (Figure 1B). These findings mean that the expression of the X-miRNAs is lower in the left testis than in the right testis, which further suggests that Y-CBS could have higher proportion in the left testis than in the right testis.

### 3.2. The Y:X Ratio Was Elevated in the Left Testis but Balanced in the Right Testis

**Figure 2:**
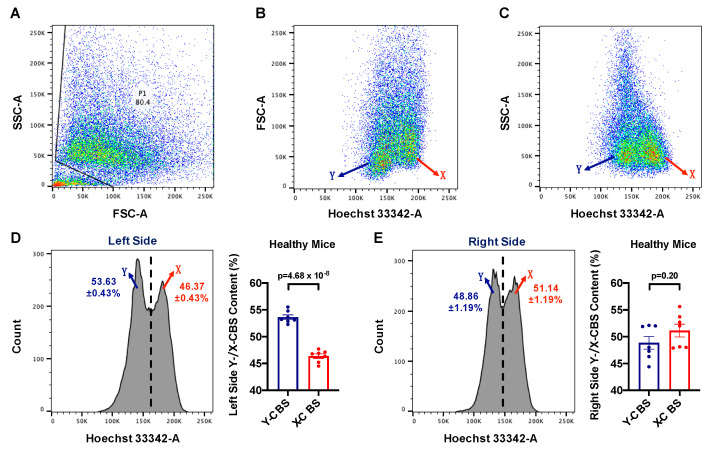
Results from flow cytometry of mice sperm stained with Hoechst 33342. Sperms were automatically sorted into X- and Y-CBS populations by flow cytometry. Representative two-dimensional dot plot side-scatter area (SSC-A) versus forward-scatter area (FSC-A) analysis of sperms collected (**A**). A dot plot displaying Hoechst 33,342 fluorescent intensity versus FSC-A (**B**) and SSC-A (**C**). Histogram displaying Hoechst 33,342 fluorescent intensity of sorted X- and Y-CBS in left (**D**) and right side (**E**) with statistical results attached (N = 7, mean ± SEM).

Spermatozoa released from testes must translocate through the epididymis for the process of sperm maturation in order to acquire the ability of fertilization, and the mature sperms are mainly stored in caudal epididymis [33]. To confirm our hypothesis as stated above, we detected the Y:X ratio in the left and right caudal epididymis with flow cytometry, which has been proved to be the most effective sperm analysis method so far [28,31]. Based on the different DNA content of the X-CBS and Y-CBS, the ratio of X-CBS and Y-CBS populations was analyzed by flow cytometry according to the same gating strategy (Figure 2A–C). We found that the proportion of Y-CBS is significantly higher than that of X-CBS (Y:X ratio = 1.16:1) in the left testis (Figure 2D, *p*-value = 4.68 × 10^−8^). However, the Y:X ratio is about 1:1 (Y:X ratio = 0.96:1) in the right testis (Figure 2E, *p*-value = 0.20). These findings support the predictive results as well. Meanwhile, we carried out the statistical comparison in a different way, comparing the ratio of left X-CBS to right X-CBS, and the ratio of left Y-CBS to right Y-CBS (see also Supplementary Figure S1).

## 4. Discussion

During mammalian spermatogenesis, primordial germ cells develop into spermatogonia, giving rise to spermatocytes that undergo two meiotic divisions to become round spermatids. Spermatogenesis is a complex process of cell differentiation controlled by many factors, among which gene regulation in the spermatogenic cells plays a pivotal role. It is well known that the Y:X ratio is 1:1 in mammal sperm under normal conditions. In this study, we first revealed that the X-miRNAs show a lower expression level in the left testis than in the right testis in normal mice, suggesting that the Y:X ratio could be unbalanced between the left testis and the right testis. We further uncovered that the Y:X ratio was significantly elevated in the left testis but balanced in the right testis using flow cytometry. Although previous studies reported biased Y:X ratio existing in specific genetic (e.g., gene mutation) and environmental (e.g., pollutants) conditions, according to our knowledge, this is the first time the interesting phenomenon of unbalanced Y:X ratio has been observed, especially in one-side testis in healthy mice. It is important to validate whether this finding is consistent when using other mammals, for example rats, dogs, and especially humans. Moreover, it is also important to explore why and how biased Y:X ratio occurs only in the left testis. We also think there may be a causal link between the X-miRNA expression level and spermatogenesis, which is being further studied and analyzed currently. Apart from that, we also considered many other directions. For example, is there any miRNA specifically expressed in one side of the epididymis or testis? Do some miRNAs related to reproduction present an unbalanced expression level? Do X- and Y-chromosome-specific genes exist in interactions and cross talks that relate to the interesting phenomenon? Finally, we believe this difference has biological significance in physiology and pathophysiology, which remains to be explored in the future.

## Data Availability

All data are available from the corresponding author upon request.

## Supplementary Materials

The following are available online at https://www.mdpi.com/article/10.3390/life11111219/s1, Figure S1: Results from flow cytometry of mice sperm stained with Hoechst 33342.
