# Supplementary material for: Y/X-Chromosome-Bearing Sperm Shows Elevated Ratio in the Left but Not the Right Testes in Healthy Mice
Source: Life (Basel). 2021 Nov 11;11(11):1219. doi: 10.3390/life11111219 (PMC8621333; doi:10.3390/life11111219)
Supplement: Supplementary file 1 [file life-11-01219-s001.zip › life-1426340-SI.pdf]

life

MDPI

*Communication*

# Y/X-Chromosome-Bearing Sperm Shows Elevated Ratio in the Left but not the Right Testes in Healthy Mice

**Chengqing Hu [1,†], Jiangcheng Shi [1,†], Yujing Chi [2], Jichun Yang [1,*] and Qinghua Cui [1,*]**

[1] Center for Noncoding RNA Medicine, Department of Physiology and Pathophysiology, Department of Biomedical Informatics, MOE Key Laboratory of Cardiovascular Sciences, School of Basic Medical Sciences, Peking University, 38 Xueyuan Rd, Beijing 100191, China; Hcq@bjmu.edu.cn (C.H.); sjc@bjmu.edu.cn (J.S.)
[2] Department of Central Laboratory & Institute of Clinical Molecular Biology, Peking University People's Hospital, Beijing 100044, China; chiyujing@bjmu.edu.cn
* Correspondence: yangj@bjmu.edu.cn (J.Y.); cuiqinghua@bjmu.edu.cn (Q.C.); Tel.: 010-82801403 (J.Y.); 010-82801001 (Q.C.)
† These authors contributed equally to this work.

**Abstract:** The sex chromosomes play central roles in determining the sex of almost all of the multicellular organisms. It is well known that meiosis in mammalian spermatogenesis produces ~50% Y- and ~50% X-chromosome-bearing sperm, a 1:1 ratio. Here we first reveal that the X-chromosome-encoded miRNAs show lower expression levels in the left testis than in the right testis in healthy mice using bioinformatics modeling of miRNA-sequencing data, suggesting that the Y:X ratio could be unbalanced between the left testis and the right testis. We further reveal that the Y:X ratio is significantly elevated in the left testis but balanced in the right testis using flow cytometry. This study represents the first time the biased Y:X ratio in the left testis but not in the right testis is revealed.

**Keywords:** X-chromosome-bearing sperm; Y-chromosome-bearing sperm; sex chromosome

**Citation:** Hu, C.; Shi, J.; Chi, Y.; Yang, J.; Cui, Q. Y/X-Chromosome Bearing Sperm Shows Elevated Ratio in the Left but not the Right Testes in Healthy Mice. *Life* **2021**, *11*, 1219. https://doi.org/10.3390/life11111219

Academic Editors: Yudong Cai and Tao Huang

Received: 1 October 2021
Accepted: 31 October 2021
Published: 11 November 2021

**Publisher's Note:** MDPI stays neutral with regard to jurisdictional claims in published maps and institutional affiliations.

**Copyright:** © 2021 by the authors. Submitted for possible open access publication under the terms and conditions of the Creative Commons Attribution (CC BY) license (https://creativecommons.org/licenses/by/4.0/).

**Supplementary Materials:**

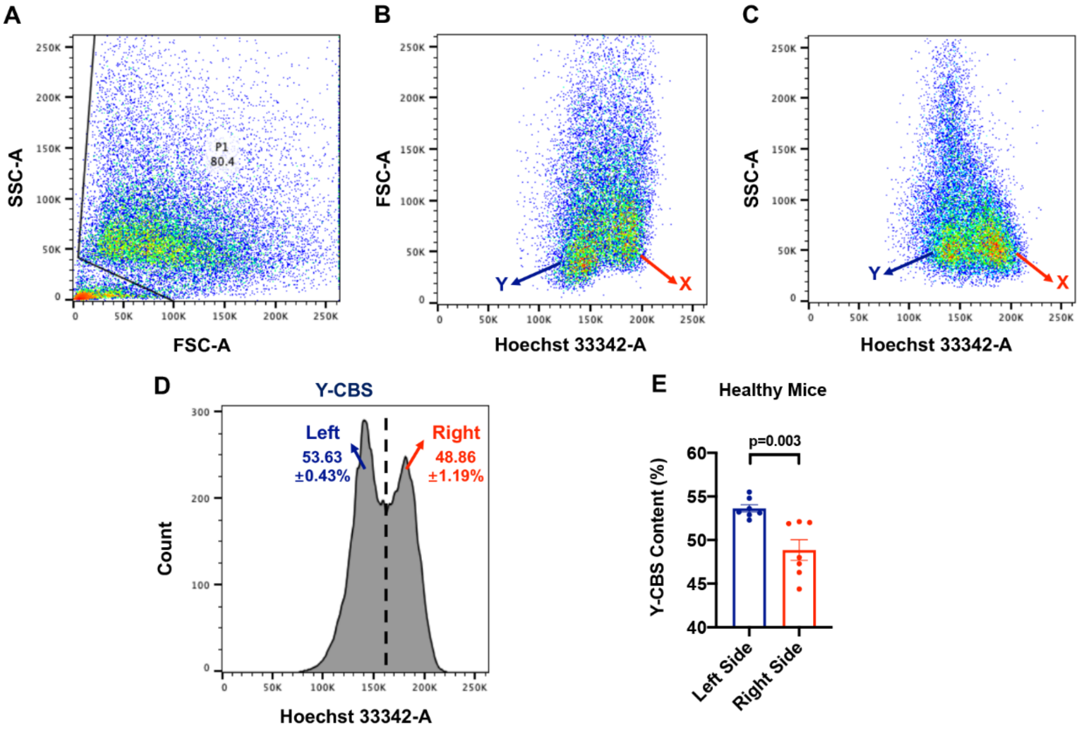


**Figure S1.** Results from flow cytometry of mice sperm stained with Hoechst 33342.
